# Coagulation Factor X Interaction with Macrophages through Its N-Glycans Protects It from a Rapid Clearance

**DOI:** 10.1371/journal.pone.0045111

**Published:** 2012-09-25

**Authors:** Mohamad Kurdi, Ghislaine Cherel, Peter J. Lenting, Cécile V. Denis, Olivier D. Christophe

**Affiliations:** 1 INSERM Unit 770, Le Kremlin-Bicêtre, France; 2 UMR_S 770, Univ Paris-Sud, Le Kremlin-Bicêtre, France; Leiden University Medical Center, The Netherlands

## Abstract

Factor X (FX), a plasma glycoprotein playing a central role in coagulation has a long circulatory half-life compared to closely related coagulation factors. The activation peptide of FX has been shown to influence its clearance with two N-glycans as key determinants of FX’s relatively long survival. To decipher FX clearance mechanism, organ biodistribution and cellular interactions of human plasma FX (pd-FX), recombinant FX (rFX), N-deglycosylated FX (N-degly-FX) and recombinant FX mutated at both N-glycosylation sites (rFX^N181A–N191A^) were evaluated. Biodistribution analysis of ^125^I-labelled FX proteins after administration to mice revealed liver as major target organ for all FX variants. Liver tissue sections analysis showed an interaction of pd-FX and N-degly-FX to different cell types. These findings were confirmed in cell binding studies revealing that FX and FX without N-glycans interact with macrophages and hepatocytes, respectively. N-degly-FX appeared to be degraded in hepatocytes while interestingly pd-FX was not by macrophages. Furthermore, the chemical inactivation of macrophages by gadolinium chloride resulted in a significant decrease of circulating pd-FX into mice and not of N-degly-FX. Altogether our data lead to the conclusion that FX interaction with macrophages through its N-glycans protects it from a rapid clearance explaining its relatively long circulatory half-life.

## Introduction

Human factor X (FX) is a vitamin K-dependent glycoprotein synthesized in the liver that circulates in plasma at a concentration of 10 µg/mL as a two-chain zymogen protein. It is composed of the light chain containing a gamma-carboxyglutamic acid-rich domain or Gla domain followed by two epidermal growth factor (EGF)-like domains linked by a disulfide bond to the heavy chain. The heavy chain contains an activation peptide and a serine-protease domain. FX plays a central role in blood coagulation. During this process, FX is activated to FXa by proteolytic cleavage of the heavy chain and subsequent release of the 52 amino acid activation glycopeptide. This cleavage also leads to a rearrangement of the serine protease domain and the formation of the catalytic site of the enzyme. Subsequently, FXa forms a high affinity macromolecular complex with other components of the prothrombinase complex: Factor Va (FVa), negatively-charged phospholipid surfaces and calcium. This prothrombinase complex activates prothrombin to thrombin leading to the formation of a hemostatic plug and local hemostasis by conversion of fibrinogen into fibrin [1]–[5].

While the functions, structure, and structure-function relationships of FX and FXa have been widely studied, the clearance process of the circulating enzyme and particularly of the zymogen has remained largely unexplored. Strikingly, FX has a long survival (half-life of 40 hours) compared to closely related vitamin K-dependent coagulation factors like factors VII (5 hours), IX (18–24 hours) and protein C (4 hours) [6]–[8]. In view of the promising therapeutic strategies based on modified FX molecules to treat hemophilia [9], [10], a better knowledge of FX clearance mechanisms appears necessary. Interestingly, FX clearance kinetics showed a slower clearance rate than that of FXa, which has a half-life reduced to only few minutes [11]. There are several structural differences between FXa and FX: among them the presence of an active catalytic site, and absence of the activation peptide in FXa compared to FX. The role of the catalytic site in FXa clearance has been investigated using inactivated FXa. Two groups showed that inactivation of the catalytic site did not prolong the half-life of FXa to the value observed of FX [11], [12] suggesting that the activation peptide domain of FX could play a role in FX clearance. This hypothesis has been investigated in two more recent studies. First, we have shown that the carboxy-terminal end of the activation peptide plays a crucial role in FX catabolism, both in recovery and half-life of the protein [13]. Moreover, it was also shown that insertion of the FX activation peptide in FVII increased the terminal half-life of the latter 4-fold [14]. Interestingly, both studies demonstrated that the two sole N-linked glycans of FX at positions 181 and 191 of its activation peptide represent important structural determinants for the recovery and half-life of the injected protein. In view of these observations, the purpose of this study was to explore the mechanism that could explain the role of the N–glycosylation sites in FX clearance. To this end, we have compared removal pathways from the circulation of human plasma and recombinant FX (pd-FX and rFX) with N-deglycosylated plasma FX (N-degly-FX) and rFX mutated at both N-glycosylation sites (rFX^N181A–N191A^), and attempted to determine organs and cell-types potentially involved in this process.

## Materials and Methods

An extensive description of materials and methods can be found in the Materials and Methods S1.

**Figure 1:**
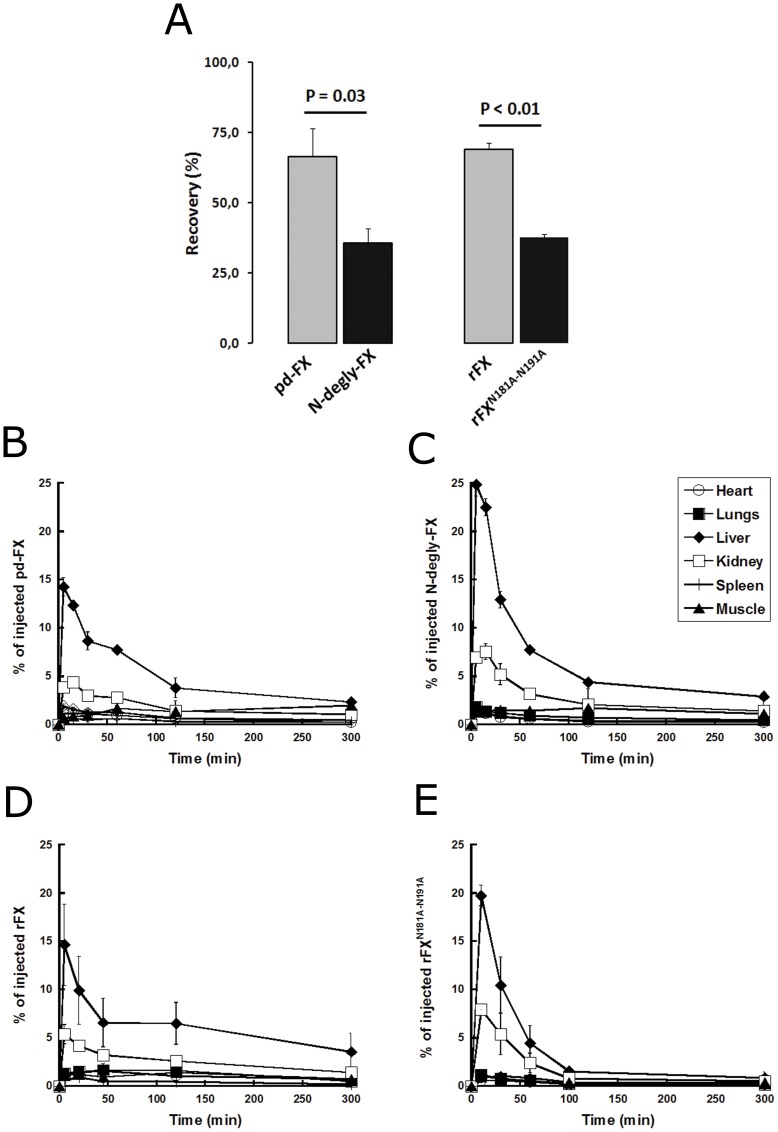
In vivo organs biodistribution and blood recovery of FX variants. (A) Five minutes after injection of I^125*−*^pd-FX, I^125*−*^N-degly-FX, rFX, or rFX^N181A–N191A^ in mice, the relative amount of radioactivity associated to blood after withdrawal was compared to the total amount injected and called recovery. Mice were injected with I^125*−*^pd-FX (B), I^125*−*^N-degly-FX (C), rFX (D), or with rFX^N181A–N191A^ (E). Associated radioactivity to several organs was counted at selected time points and the relative amount of radioactivity compared to the total amount injected was calculated. Three mice were used for each time point. Data represent mean triplicate values ± S.D.

### Mice

Wild-type mice C57Bl/6 were purchased from Janvier (Le Genest Saint Isle, France). Housing and experiments were done as recommended by French regulations and the experimental guidelines of the European Community. The Animal Care and Use Committee of INSERM approved animal experiments (licence #B-94-043-13).

**Figure 2:**
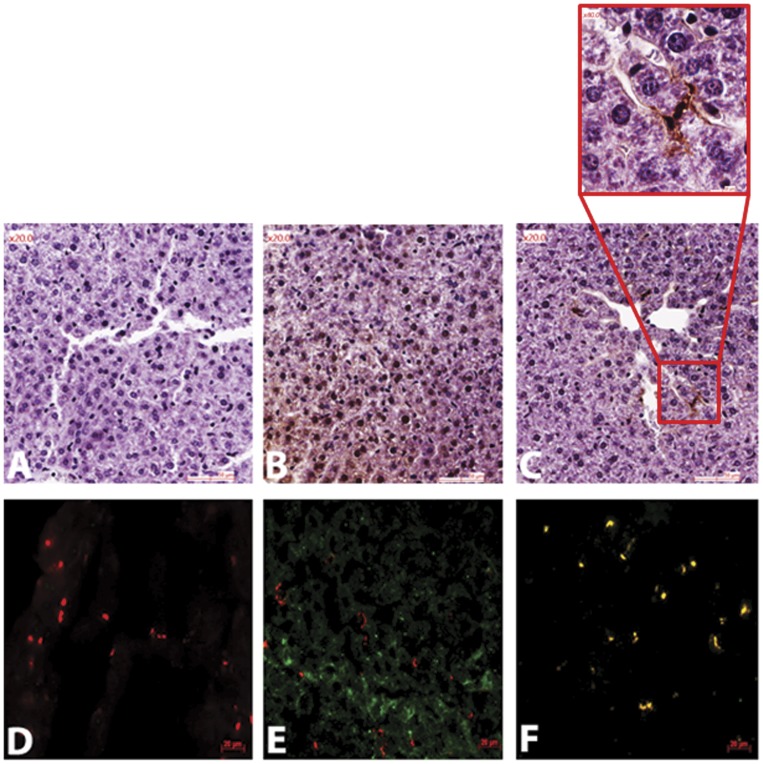
Identification of cells as main target for FX variants in liver. Liver sections from mice injected with 20 µg/mouse of pd-FX (C, F), N-degly-FX (B, E) or with PBS as control (A, D). Livers were collected 10 minutes after injection. In a first set of sections (A, B, and C) FX variants are stained with rabbit anti-human FX (Eurogentec followed by highly sensitive polymer-based detection reagent (HRP) (Dako, Trappes, France) and then with diaminobenzamidine. These tissue sections were slightly counterstained with diluted hematoxylin (magnification 200). One representative experiment out of six performed with different mice is shown. (D, E, and F) Merged images of liver sections stained with rabbit anti-human FX followed by Alexa Fluor 488 F(ab’)_2_ fragment (Invitrogen) of goat anti-rabbit IgG (H+L) as secondary antibody (green) and with monoclonal rat anti-mouse CD68 (AbD Serotec, Oxford, UK) followed by TRITC-conjugated goat anti-rat immunoglobulins (Southern Biotech, Birmingham, AL, USA) (red). Original magnification 200. One representative experiment out of four performed with different mice is shown.

### Factor X Protein Preparation

Full-length Glycosidase digestion experiments under mild conditions on N-linked glycans of *plasma-derived factor X (pd-FX from Haematologic Technologies Inc, Vermont, USA)* were carried out using *PNGase F (N-Glycosidase F from New England Biolabs Inc, Essex Junction, MA, USA)* from Chryseobacterium as previously described [15]. Protein purity (>95%) and full removal of N-glycans was assessed using 15% SDS-polyacrylamide gel electrophoresis analysis *(NuPAGE Bis-Tris gel from Invitrogen, Cergy-Pontoise, France)* under non-reducing conditions followed by staining with Coomassie Brilliant Blue R-250 (Fig. S1).

**Figure 3:**
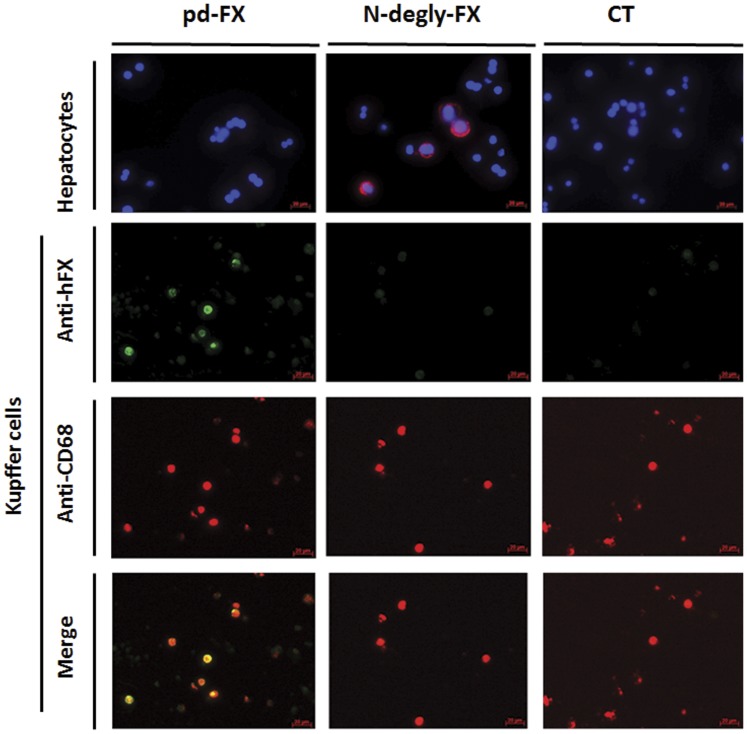
In vitro binding of FX variants to purified mouse liver cells. Purified murine hepatocytes or Kupffer cells were incubated with 10 µg/mL of pd-FX, N-degly-FX or PBS as control (CT) for 1 h at 4°C. For purified hepatocytes, nucleus/DNA was stained with DAPI (blue) and FX variants were visualized by red fluorescence using mouse monoclonal antibody and rabbit polyclonal antibody both anti-human FX in Duolink assay. Purified Kupffer cells were stained with rabbit anti-human FX followed by Alexa Fluor 488 F(ab’)_2_ fragment of goat anti-rabbit IgG (H+L) as secondary antibody (green) and with monoclonal rat anti-mouse CD68 followed by TRITC-conjugated goat anti-rat immunoglobulins (red). Original magnification 630.

Recombinant wild-type FX (rFX) and FX mutated at both N-glycosylation sites (rFX^N181A–N191A^) were constructed, produced in BHK-21 cells *(ATCC, Molsheim, France)* and purified as reported [13], [16]. rFX^N181A–N191A^ corresponds to the FX/AP^176–194-N181A–N191A^ and thus lacks the C-terminus of the activation domain.

**Figure 4:**
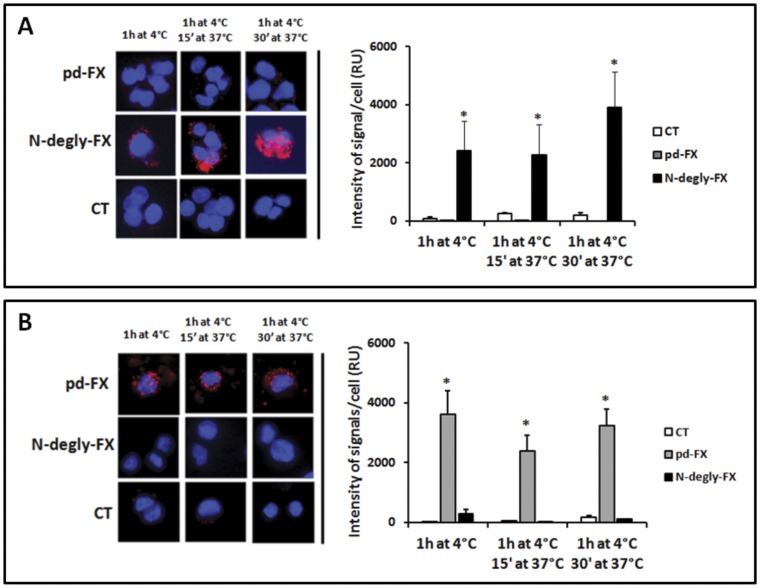
Binding and internalization of pd-FX by differentiated THP-1 and N-degly-FX by HepG2 cells. (A) HepG2 cells and (B) THP-1 cells differentiated to macrophages by PMA (see Materials and Methods) were incubated with 10 µg/mL of pd-FX, N-degly-FX or with PBS as control (CT) for 1 h at 4°C. Then, after washing cells were incubated at 37°C for 15 and 30 min. Nucleus/DNA was stained with DAPI (blue) and FX variants were visualized by red fluorescence using mouse monoclonal and rabbit polyclonal antibodies both anti-human FX in a proximity ligation assay (PLA)–based method. Fluorescence intensity was quantified using BlobFinder v3.2 software package. Data represent the mean fluorescent intensity ± S.D. of 20–35 cells, obtained from three independent experiments.

Pd-FX, N-degly-FX, rFX and rFX^N181A–N191A^ were labeled using Na^125^I (Perkin-Elmer, Waltham, MA, USA) and Iodo-Gen (Pierce Chemical Co, Rockford, IL, USA) [17]. Specific radioactivities varied from 0.1 to 5 µCi.µg*^−^* ^1^.

### Pharmacokinetics and Biodistribution of Radiolabeled FX Variants

21 females mice, 8 weeks old were used. 10 µg of purified ^125^I-pd-FX, ^125^I-N-degly-FX, ^125^I-rFX or ^125^I-rFX^N181A–N191A^ diluted in phosphate buffer saline (or PBS from PAA laboratories, Pasching, Austria) were injected per mouse via the tail vein. At different time-points (from 5 to 300 minutes) after injection, mice were anesthetized with tribromoethanol (0.15 mL/g body weight), blood was collected by retro-orbital venous sampling on citrate, and organs (heart, liver, kidneys, lungs, spleen and muscles) were collected immediately after blood withdrawal and sacrifice of the mice. Organs were removed and weighed on a microbalance sensitive to 0.001 mg (AT261 Delta Range, Mettler-Toledo Inc., Columbus, OH, USA) and recorded. Three mice were used for each time point. Radioactivity in blood, plasma and organs was measured in a gamma counter, and the percentage of radioactivity present in each tissue was calculated in relation to the total amount of injected radioactivity.

**Figure 5:**
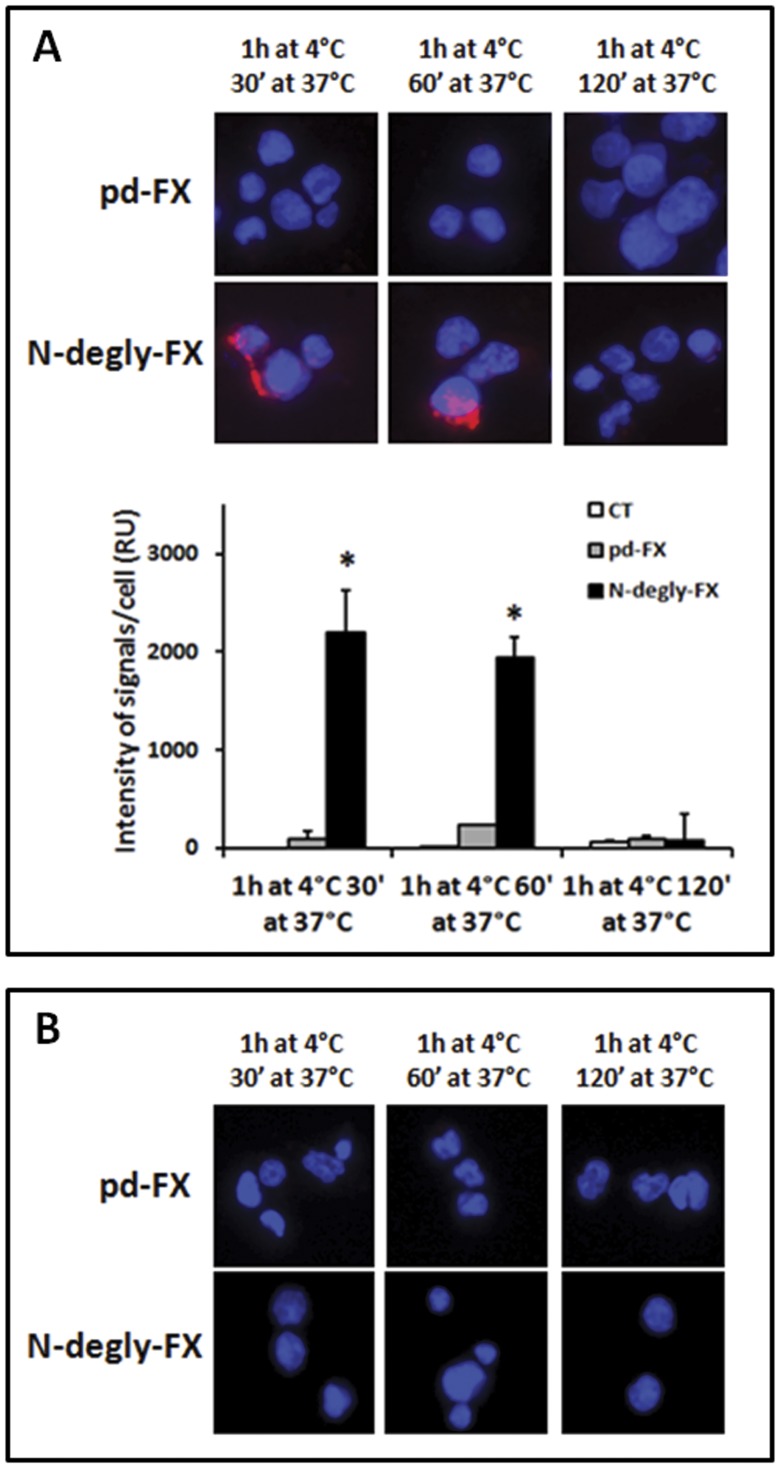
Investigation of the co-localization of pd-FX and N-degly-FX with early endosomes in differentiated THP-1 cells and HepG2 cells. (A) HepG2 cells and (B) THP-1 cells differentiated to macrophages by PMA (see Materials and Methods) were incubated with 10 µg/mL of FX variants for 60 min at 4°C then washed and incubated for 30, 60 and 120 min at 37°C. Nucleus/DNA was stained with DAPI (blue) and FX variants were co-localized by red fluorescence using goat anti-human FX with anti-early endosome-antigen 1 in a proximity ligation assay (PLA)–based method. A representative set of experiments is presented. Fluorescence intensity was quantified using BlobFinder v3.2 software package. Data represent the mean fluorescent intensity ± S.D. of 20–35 cells, obtained from three independent experiments.

**Figure 6:**
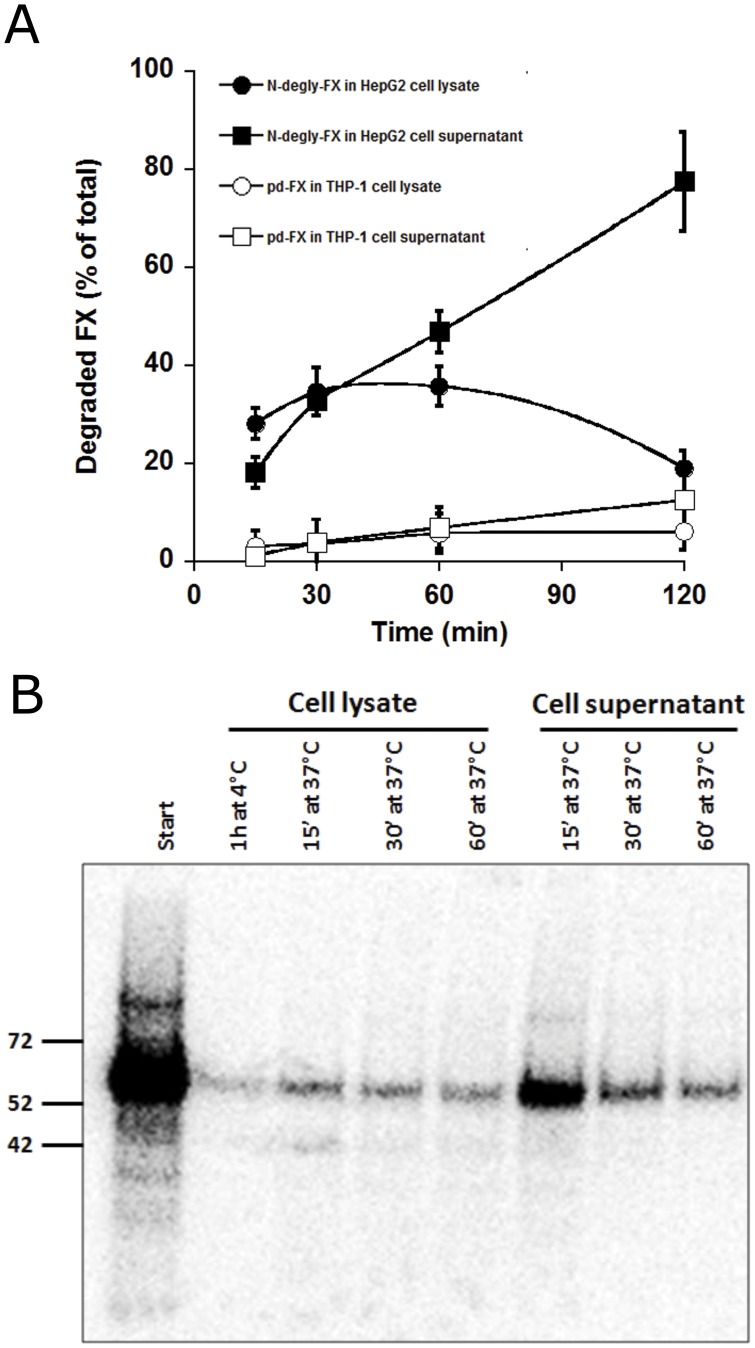
Investigation of ^125^I-pd-FX and ^125^I-N-degly-FX degradation by differentiated THP-1 cells and HepG2 cells, respectively. (A) ^125^I-pd-FX and ^125^I-N-degly-FX were added to differentiated THP-1 cells and HepG2 cells, respectively, for a 1-hour period at 4°C. Cells were then washed, and incubation was continued at 37°C to initiate endocytosis. At indicated time points, cell lysates and cell supernatants were taken to determine the amount of degraded material. Degraded material is defined as the radioactivity that is soluble in 10% trichloroacetic acid. In all experiments, controls were included to determine the amount of nonspecific degradation in the absence of cells, which routinely was less than 10% of degradation in the presence of cells. Data represent mean ± S.D. of 3 experiments. (B) ^125^I-pd-FX was incubated with differentiated THP-1 cells at indicated time points (1h at 4°C, then for 15′, 30′ and 60′ at 37°C). Cell lysate and supernatant were migrated on SDS-PAGE and results were visualized by autoradiography using PharosFX™ Plus Molecular Imager (BioRad, Hercules, CA, USA).

### Tissue Collection

Mice were injected intravenously in the tail-vein with pd-FX, N-degly-FX, rFX or rFX^N181A–N191A^ diluted at 10 µg/mL in PBS. Mice were bled, anesthetized and sacrificed. Then, tissue was collected and perfused with PBS. For paraffin sections, individual lobes of mice livers were embedded in paraffin wax blocks. For cryostat sections, mice livers were perfused with PBS, embedded in Tissue-Tek OCT-compound (miles laboratories, Elkhart, IN), and immediately frozen in liquid nitrogen and maintained at −20°C. Sections were washed in PBS and permeabilized with triton 0.5% in PBS and were incubated with bovine serum albumin 3% (BSA) in PBS containing murine IgG (1/500 = 2.5 µg/mL) to saturate non-specific section surfaces and Fc receptors.

**Figure 7:**
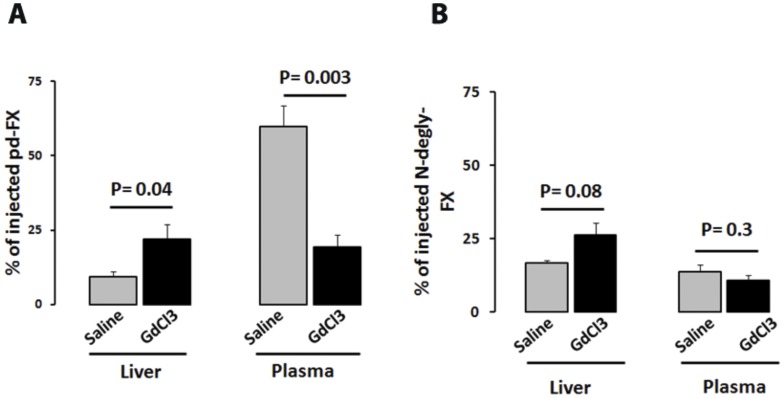
Decreased pd-FX levels upon gadolinium chloride treatment. Mice were treated with saline or GdCl_3_, and 24 hours after treatment ^125^I-pd-FX or ^125^I-N-degly-FX was administered. Then, 15 minutes later plasma samples were taken and the associated radioactivity was counted. The values obtained were compared to the total amount injected and expressed as percent. At the same time point, mice were anesthetized, sacrificed and their liver was taken and washed with PBS. The associated radioactivity was compared to the total amount injected. Data represent mean ± S.D. of 3 experiments.

### Gadolinium Chloride Treatment of Mice

The experiment was done as previously described [18]. Briefly, Gadolinium chloride (GdCl_3_, 50 mg/kg of body weight) diluted in 200 µl physiological serum (or 200 µl physiological serum as control) was given to mice via intravenous tail injection 24 hours prior to the administration of 10 µg of purified ^125^I-pd-FX or ^125^I-N-degly-FX diluted in PBS.

### Cell Culture

The human hepatocellular carcinoma HepG2 cell line (ATCC, Molsheim, France) was used at passage lower than 5; cells were cultured in Dulbecco’s modified Eagle’s medium (DMEM/Ham’s F-12 with stable Glutamine from PAA laboratories), supplemented with 10% fetal bovine serum (FBS) and 1% penicillin/streptomycin within a CO_2_-incubator at 37°C with 5% CO_2_. Cells were maintained in T-25 flasks, and re-plated in 24-well tissue culture plates. Binding, endocytosis, or degradation studies were performed within 24 h after reaching confluence.
The human umbilical vein endothelial cells (or HUVECs from PromoCell, Heidelberg, Germany) were used at passage lower than 3; they were cultured in endothelial cell growth medium, supplemented with supplement kit containing all growth factors and serum necessary for HUVECs amplification (PromoCell). Binding, endocytosis or degradation studies were performed within 24 h after reaching confluence.
The human acute monocytic leukaemia cell line (THP-1) from ATCC was used at passage lower than 5; cells were maintained at the concentration of 10^6^ cell/mL in RPMI medium (Invitrogen) supplemented with 10% FBS, 2 mM GlutaMax™, 1% sodium pyruvate, 1% nonessential amino acids, 1% penicillin/streptomycin, and β-mercaptoethanol (20 µM). Cells were differentiated with 100 nM phorbol myristate acetate (PMA) (Sigma) during 24 h in 24-well tissue culture plates [19]. These cells are phenotypically characterized by low expression of surface markers CD4 and CD64 and high expression of CD36 and CD11b.

### Protein Binding and Internalization by Isolated Cells

Binding studies were performed at 4°C and internalization at 37°C in 24-well tissue culture plates. Cells were washed once with PBS and maintained in serum free medium for 1 h at 37°C. Then, medium was changed with 500 µL of cold serum free medium and FX proteins were added at final concentration of 10 µg/mL. For binding studies, cells were incubated for 1 h at 4°C, then cells were washed thrice with PBS, and fixed with paraformaldehyde (PFA) 4%. For internalization study, after incubation at 4°C, cells were washed and incubated for 0.25, 0.5 and 1 h within an incubator at 37°C with 5% CO_2._ Finally, cells were fixed as in binding studies, and permeabilized with 0.5% triton X-100.

### Immunostaining of Protein Binding and Internalization by Isolated Cells

Binding and internalization results were visualized by classical immunofluorescence or immunofluorescence using a proximity ligation assay (PLA)–based methodology (Olink Bioscience, Uppsala, Sweden). Briefly, cells were incubated with bovine serum albumin 3% (BSA) in PBS containing goat IgG (1/1000 = 2.5 µg/mL) to saturate non-specific section surfaces and Fc receptors. For classical immunofluorescence, rabbit anti-human FX (1∶1000 dilution = 3.37 µg/mL) (Eurogentec, Angers, France) was used as primary antibody, followed by Alexa Fluor 488 F(ab’)_2_ fragment of goat anti-rabbit IgG (H+L) as secondary antibody (Invitrogen). For the PLA–based methodology, two primary antibodies (2 µg/mL) raised in different species recognizing the target antigens of interest were used, either mouse anti-human FX (prepared in our laboratory using purified pd-FX as immunogen) with rabbit anti-human FX (Eurogentec) or goat anti-human FX (CEDARLANE Laboratories Inc, Burlington, NC, USA) with rabbit anti-early endosome-antigen 1 (EEA1) (Abcam, Cambridge, UK). Species-specific secondary antibodies conjugated with a short DNA probe were added and incubated for 2 h in humid-chamber at 37°C. When the PLA probes are in close proximity (<40 nm), the DNA strands can interact through a subsequent addition of two other circle-forming DNA oligonucleotides. After the amplification reaction, several-hundredfold replication of the DNA circle has occurred, and labelled complementary oligonucleotide probes highlight the product. The resulting high concentration of fluorescence in each single-molecule amplification product is easily visible as a distinct bright red dot when viewed with a fluorescent microscope.

### Binding, Internalization, and Degradation of ^125^I-FX Variants by Isolated Cells

Radiolabeled FX variants were incubated with cultured cells for binding, internalization and degradation study, as described above. To investigate the presence of degradation products in the supernatant, supernatants were taken at different time points. For evaluation of the binding on the cell surface, or of internalization and degradation of protein inside the cells, cells were treated with triton 1% for 1 h. Samples were migrated on SDS-PAGE and results were visualized by autoradiography using PharosFX™ Plus Molecular Imager (BioRad, Hercules, CA, USA). In another set of experiments, cell lysates and cell supernatants were taken to determine the amount of degraded material. Degraded material is defined as the radioactivity that is soluble in 10% trichloroacetic acid. In all experiments, controls were included to determine the amount of nonspecific degradation in the absence of cells.

## Results

### Plasma Recovery and Organ Biodistribution of ^125^I-pd-FX, ^125^I-N-degly-FX, ^125^I-rFX, and ^125^I-rFX^N181A–N191A^

Our first objective was to investigate the importance of N-glycosylation sites of FX on its plasma recovery and tissue distribution. Plasma recovery of ^125^I-pd-FX and ^125^I-rFX 5 min after administration to mice was with similar values of 66±10% and 69±2%, respectively, whereas it was only 36±5% and 38±1% for ^125^I-N-degly-FX and ^125^I-rFX^N181A–N191A^, respectively (Fig. 1A). These data indicate that the presence of N-glycans on FX influences its recovery. Biodistribution of ^125^I-pd-FX, ^125^I-N-degly-FX, ^125^I-rFX, and ^125^I-rFX^N181A–N191A^ was evaluated in mice organs over a 6 hours period (Fig. 1). Liver was the major target organ for all molecules. However, a significant different peak value in this organ was observed after 5 min of administration for pd-FX and N-degly-FX, with 14±1% *versus* 25±1% (p = 0.001), respectively. A similar observation was made after 10 min administration for rFX and rFX^N181A–N191A^, with 13±4% *versus* 20±1% (p<0.05), respectively. Apart from liver, we found that also kidney takes up efficiently pd-FX (16±1% and 14±1% FX/mg tissue for kidney and liver, respectively), N-degly-FX (28±5% and 23±1% N-degly-FX/mg tissue for kidney and liver, respectively), r-FX (20±4% and 13±4% FX/mg tissue for kidney and liver, respectively) and rFX^N181A–N191A^ (34±3% and 20±1% % N-degly-FX/mg tissue for kidney and liver, respectively). However, given the respective sizes of the liver and kidney with 967±114 mg *versus* 230±14 mg (p<0.0001; n = 21 mice), the absolute contribution of kidney compared to liver remains limited (Fig. 1). The other organs studied such as spleen, heart, and lungs did not take up significant amounts of pd-FX, N-degly-FX, rFX, or I-rFX^N181A–N191A^.

### Identification of Liver Cells Targeting FX Variants in vivo

The observation that the liver is the main organ responsible for the clearance of circulating pd-FX, N-degly-FX, rFX, or rFX^N181A–N191A^ (Fig. 1) led us to identify the cellular destination of these proteins in this organ. To this end, mouse liver was isolated 10 minutes after injection of FX variants. Liver tissue sections were prepared for immuno-histochemical analysis to reveal the localization of each variant on liver cell types. As controls, liver sections of mice injected with 200 µL of PBS were used, which all were negative for FX (Figs. 2A & D). For liver tissue sections obtained from N-degly-FX injected mice, the localization of FX was predominantly to hepatocytes, which is the main cell type in the liver (Fig. 2B). In contrast, liver tissue sections from pd-FX injected mice did not show any presence of FX in hepatocytes but rather on sinusoidal cells, probably Kupffer cells (Fig. 2C). Co-staining experiments for FX and Kupffer cells using another series of liver tissue sections demonstrated that the majority of pd-FX co-localizes with these macrophages (Fig. 2F) whereas no co-localization was observed between N-degly-FX and Kupffer cells (Fig. 2E). As controls, liver sections of mice injected with 200 µL of PBS were used, which were all negative for FX and only positive for Kupffer cells (Fig. 2E). These results suggest that different liver cell types are responsible for the uptake of pd-FX and N-degly-FX.

### In vitro Targeting of pd-FX and N-degly-FX to Isolated Mouse Liver Cells

To further identify the cellular type in the liver targeted by pd-FX and N-degly-FX, murine liver cells were isolated and tested in vitro for their capacity to bind either pd-FX or N-degly-FX. The cells were incubated with the proteins for 1 hour at 4°C to avoid endocytosis. As shown in Figure 3, this approach revealed that pd-FX binds to isolated Kupffer cells but not to isolated hepatocytes. In contrast, no binding of N-degly-FX to isolated Kupffer cells was observed, whereas the protein showed efficient binding to isolated hepatocytes.

### Evaluation of pd-FX, N-degly-FX, rFX, and rFX^N181A–N191A^ Binding to Human Cell Lines

The observation that pd-FX and N-degly-FX were targeted to Kupffer cells and hepatocytes, respectively, led us to test the ability of the two FX variants and their corresponding recombinant to bind to different human cell lines in vitro. Therefore, pd-FX, N-degly-FX, rFX, and rFX^N181A–N191A^ were incubated with HepG2 cells (hepatocytes) or differentiated THP-1 (macrophages) for 1 h at 4°C to study their capacity to bind these cells. As shown in Figure 4, background staining was observed for the negative control (PBS), whereas distinct fluorescent signals were detected for N-degly-FX incubated with HepG2 cells and for pd-FX with differentiated THP-1. In quantitative terms, significantly more fluorescence intensity was detected for these incubations than for the negative control (p<0.05; Figs. 4A & 4B). A similar pattern of binding was observed for the recombinant counterparts, with rFX binding to THP-1 cells but not to HepG2 cells, and rFX^N181A–N191A^ binding to HepG2 cells but not to THP-1 cells (Fig. S2). Thus, these data are compatible with a dissimilar cellular targeting between normal FX and FX that lacks the N-linked glycans in the activation peptide.

### Evaluation of FX Variants Internalization and Degradation by Human Cells

Subsequent experiments aimed to investigate whether the different cell types were able to endocytose and degrade pd-FX or deglycosylated derivative. When N-degly-FX bound to HepG2 cells was incubated at 37°C, we could observed a diffusion of the fluorescent signal from the cell surface to the cytoplasm (Fig. 4A). This suggests that these cells take up at least part of the protein. This possibility was confirmed by the detection of a co-localization between N-degly-FX and the early endosomal marker EEA-1 (Fig. 5A). Interestingly, this co-localization disappeared gradually during the time course of incubation and was virtually absent after 2 hours at 37°C (Fig. 5A; Fig. S3). One option that could explain the loss of signal is that the endocytosed N-degly-FX is degraded within the HepG2 cells. We tested this possibility by measuring the fraction of radioactive protein that remains in solution following precipitation with 10% trichloroacetic acid (TCA). This soluble fraction is a measure for the amount of degraded protein. In time, we observed a concomitant increase in the amount of degraded N-degly-FX both in the cell lysate and supernatant, with virtually all protein being degraded after 2 H (Fig. 6A). Thus, it seems that N-dely-FX is efficiently bound by HepG2 cells, and subsequently targeted to intracellular degradation compartments.

As for pd-FX, we performed similar experiments using THP-1 cells, since no binding to HepG2 cells was observed. In contrast to N-degly-FX, no change in cellular staining was observed upon incubation of the THP-1 cells at 37°C, suggesting that pd-FX was not redistributed into the cell (Fig. 4B). Indeed, no co-localization of pd-FX with EEA-1 could be detected, nor was there any significant increase in radiolabeled pd-FX degradation fragments in the lysate or supernatant (Figs. 5B and 6A). In order to test whether the protein indeed remains intact while being incubated with THP-1 cells, we analyzed both the supernatant and the cell-bound fraction of radiolabeled pd-FX via SDS-Page. As shown in Figure 6B, this analysis revealed that FX remains intact while being incubated with THP-1 cells rather than being degraded.

### Degradation Products of pd-FX in Mouse Plasma

To investigate whether or not degraded pd-FX can be observed in the circulation after its administration, mouse plasma was taken at different time points after ^125^I-pd-FX injection intravenously. Samples were migrated on SDS-PAGE and associated radioactivity was visualized by autoradiography. We found no evidence that pd-FX is degraded in mouse plasma up to 24 h after its injection. Only an intact band was detected over time (Fig. S4).

### GdCl_3_-induced Macrophage Reduction Alters pd-FX Catabolism

In an attempt to further explore the contribution of macrophages to the catabolism of pd-FX in vivo, mice infused with ^125^I-pd-FX or ^125^I-N-degly-FX (10 µg/mouse) were treated with saline (control) or GdCl_3_ (50 mg/kg). GdCl_3_ is an agent known to efficiently reduce the number and function of macrophages [20]–[22]. Indeed, as previously reported [18], analysis of livers taken from mice 24 hours after treatment with GdCl_3_ revealed an increase in endogenous VWF antigen levels in wild-type mice (VWF:Ag = 184±98% and 434±99% for GdCl_3_-treated mice before and 24 hours after treatment, respectively; p = 0.006; Fig. S5A) associated with a significant reduction of the number of CD68^+^ Kupffer cells (Fig. S5B). Plasma recovery of ^125^I-N-degly-FX 15 min after administration to saline- or GdCl_3_-treated mice was with similar values of 14±3% and 11±2%, respectively (p-value = 0.3; Fig. 7B). In contrast, the survival of ^125^I-pd-FX in GdCl_3_-treated mice was strongly reduced when compared to saline-treated mice with values of 20±4% and 60±7%, respectively (p = 0.003; Fig. 7A). Thus, our data point to macrophages as physiological relevant elements modulating the clearance of pd-FX, but not of its N-deglycosylated counterpart.

## Discussion

The structure of the N-linked complex-type carbohydrate side chains of plasma glycoproteins is well conserved suggesting an important role in several physiological processes such as function, antigenicity and circulatory lifetime. The aim of the present study was to address the clearance mechanism(s) of FX and how this process is modulated by the N-glycans of the protein, which are present on its activation peptide (AP). By using radiolabeled proteins, we observed that both plasma derived FX (pd-FX) and its N-deglycosylated derivative (N-degly-FX) were mainly targeted to the liver. The same observation was made with their recombinant counterparts, rFX and rFX mutated at its N-glycosylation sites (rFX^N181A–N191A^) (Fig. 1). Interestingly, the amount of N-degly-FX that was accumulated in the liver after 5 min was considerably increased compared to the amount of pd-FX (25±1 *versus* 14±1%). These data correspond to the observation that almost 2-fold higher levels of pd-FX remained in the circulation compared to N-degly-FX (67±10 *versus* 36±5%). Similar findings were obtained when recombinant materials were compared *i.e.* rFX and rFX^N181A–N191A^ (Fig. 1). This increased clearance of the N-degly-FX variant is fully in line with our previous observations, which demonstrated that a mutant of FX lacking both N-linked glycosylation sites at positions 181 and 191 in the AP displayed increased clearance in comparison to wild-type FX [13]. Apparently, the presence of N-glycans in the FX AP is pertinent to the survival of the protein. It should be noted that our previous study revealed that the carboxy-terminal region of 19 residues of the FX AP, which includes both N-glycosylation sites, is sufficient to preserve a normal survival of the protein [13]. Moreover, Johansson et al demonstrated that insertion of FX AP into the sequence of other vitamin K-dependent proteases substantially extends their half-lifes [14]. Taken together, these observations not only emphasize the importance of the N-glycosylated part of the FX AP in the survival of the protein, but also show that FX AP can be used as a tool to increase the mean circulating time of plasma proteins and therefore improve their therapeutic potential.

When we studied the biodistribution of pd-FX and N-degly-FX in the liver in more detail, we made the intriguing observation that these proteins were distributed in a dissimilar manner. We found that pd-FX accumulated into cells that probably represent Kupffer cells, whereas N-degly-FX had been taken up by hepatocytes (Fig. 2). This distinct in vivo biodistribution was confirmed in two different in vitro tests. First, binding of pd-FX and N-degly-FX to Kupffer cells and hepatocytes, respectively, was confirmed in binding experiments using cells that were freshly isolated from mouse liver (Fig. 3). Second, experiments with human cell lines, HepG2 representing hepatocytes and differentiated THP-1 representing Kupffer cells, revealed similar results (Fig. 4). A similar discrepancy of cell targeting was observed for rFX and rFX^N181A–N191A^ (Fig. S2). Our observation that pd-FX did not bind to hepatocytes is in full agreement with a previous study showing that activated FX but not its inactive zymogen interacts with HepG2 cells [23]. On the other hand, our observation that pd-FX did not bind to hepatocytes seems at odds with reports showing that FX promotes Adenovirus serotype-5 (Ad5) binding to hepatocytes [24]–[27]. One possibility that may explain this apparent contradiction is that complex formation between pd-FX and Ad5 masks or changes the orientation of N-glycans on the glycoprotein, which promotes the binding of the complex to hepatocytes. Alternatively, Ad5 itself may mediate binding to hepatocytes, a process that is facilitated by its interaction with the pd-FX. Of note, we could not observe an interaction between pd-FX and endothelial cells from human umbilical vein or freshly isolated from mouse liver (data not shown). This seems to be in contradiction with other studies where FX was suggested as able to bind to endothelial cells [28]–[30]. However, a recent study convincingly showed that FX binding to endothelial cells appeared insignificant at physiological concentrations [31], and seems to involve cell signalling [29], [30] rather than clearance of the protein.

Apart from different preferences for cellular binding, pd-FX and N-degly-FX were also different in a second manner. N-degly-FX seems to follow a regular pathway of uptake and degradation by hepatocytes as shown in Figs. 4, 5, 6. Intriguingly, this does not seem to be true for the pd-FX interaction with Kupffer cells. Even after prolonged incubation at 37°C, pd-FX remained visible at the cell surface of THP-1 macrophages and no co-localization with the early endosomal marker EEA-1 could be observed (Figs. 4 and 5). Moreover, no FX degradation products could be detected (Fig. 6). Indeed, analysis of THP-1 lysates and supernatants revealed an intact FX protein when assessed via SDS-page (Fig. 6). We also observed no degraded pd-FX in the circulation after its intravenous injection to mouse (Fig. S4). These data strongly suggested that Kupffer cells do not metabolize the pd-FX but rather are acting as storage cells for the glycoprotein and thereby reduce its clearance rate.

This notion was supported by our observation that inactivation of Kupffer cells by GdCl_3_ induces a reduction of pd-FX recovery rate at 15 min (19.4% *versus* 59.9% without GdCl_3_, p = 0.003; Fig. 7) to a level that is similar to that of N-degly-FX (p = 0.84) and an increase of its clearance rate (mean residence time of approximately 60 min *versus* 380 min without GdCl_3_). Thus, in mice treated with GdCl_3_, pd-FX is cleared more rapidly. The mechanism by which GdCl3-mediated corruption of the macrophages promotes increased clearance of pd-FX remains to be determined as well as the cell type that takes up pd-FX under these conditions.

In the present study we have established that the presence of N-glycosylation is very important in the biodistribution process of FX and constitutes a key determinant allowing an interaction of the protein with Kupffer cells. This interaction does not lead the protein to a degradation pathway but rather to a protective pathway. This process may mimic a protein-recycling program as observed for the storage pathways for immunoglobulin G and albumin that use the neonatal Fc receptor (FcRn) for transient storage in endothelial cells [32], [33]. This process could explain the long half-life of FX, especially compared to its N-deglycosylated variant as previously observed [13]. The precise mechanism and especially receptors and cell pathways involved in the process of FX elimination remain to be investigated. A mechanism with several steps involving Kupffer cells, modification of the protein, and transfer to the hepatocytes where the protein is degraded and eliminated is a possible process which has been demonstrated for another protein, the carinoembryonic antigen. [34].

The precise characterization of the clearance pathway of FX will be needed since promising therapeutic strategies based on modified FX molecules to treat haemophilia are emerging [9], [10]. Furthermore, this knowledge will constitute important value to decipher an original mechanism of clearance and could constitute the basis for the bioengineering of new therapeutic agents able to modulate the survival of a protein in the circulation.

## Supporting Information

Figure S1 **SDS PAGE Analysis of pd-FX, FXa, and N-degly-FX.** Purified proteins (2 µg/lane) were subjected to 15% SDS-polyacrylamide gel electrophoresis analysis under non-reducing conditions followed by staining with Coomassie Brilliant Blue R-250. Standards with apparent molecular weights are indicated on the left of the gel; Lane 1, pd-FX; lane 2, rFX; lane 3, N-degly-FX; and lane 4, rFX^N181A–N191A^, and lane 5 FXa. N-degly-FX was obtained after digestion of pd-FX by PNGase F. rFX and rFX^N181A–N191A^ contain at their carboxy-terminal end a supplementary sequence (EQDDPRLIDGK) recognized by monoclonal antibody HPC4 as previously reported [13], [16]. rFX^N181A–N191A^ corresponds to the FX/AP^176–194-N181A–N191A^ and thus lacks the C-terminus of the activation domain [13].
(TIF)

Figure S2 **Binding and internalization of rFX^N181A–N191A^ by HepG2 cells and rFX by differentiated THP-1.** HepG2 cells and THP-1 cells differentiated to macrophages by PMA (see Materials and Methods) were incubated with 10 µg/mL of rFX, rFX^N181A–N191A^ or with PBS as control (CT) for 1 h at 4°C. Then, after washing cells were incubated at 37°C for 15 and 30 min. Nucleus/DNA was stained with DAPI (blue) and FX variants were visualized by red fluorescence using mouse monoclonal and rabbit polyclonal antibodies both anti-human FX in a proximity ligation assay (PLA)–based method.
(TIF)

Figure S3 **Investigation of the co-localization of pd-FX and N-degly-FX with early endosomes in HepG2 cells busing high-resolution confocal images.** HepG2 cells were incubated with 10 µg/mL of pd-FX, N-degly-FX or with PBS as control (CT) for 1 h at 4°C. Then, after washing cells were incubated at 37°C for 30, 60 and 120 min at 37°C. Nucleus/DNA was stained with DAPI (blue) and FX variants were co-localized by red fluorescence using goat anti-human FX with anti-early endosome-antigen 1 in a proximity ligation assay (PLA)–based method.
(TIF)

Figure S4 **Investigation of radiolabeled FX variants degradation in mouse plasma.** Mice were injected with either (A) ^125^I-pd-FX or (B) ^125^I-N-degly-FX (10 µg/mouse) and at different time points (5, 30, 60, 120, 240, and 1440 minutes) blood samples were taken. Plasma samples were migrated on 15% SDS-polyacrylamide gel electrophoresis analysis under non-reducing conditions. Results were visualized by autoradiography using PharosFX™ Plus Molecular Imager (BioRad, Hercules, CA, USA).
(TIF)

Figure S5 **Increased of endogenous VWF levels upon gadolinium chloride treatment.** (A) Mice were treated with saline or GdCl_3_, and 24 hours after treatment (T24) endogenous VWF (mvWF) was measured by ELISA and compared to mvWF levels before (T0) saline or GdCl_3_ treatment. Data represent mean ± S.D. of 3 experiments. (B) Liver sections of wild-type mice treated with saline (normal liver) or GdCl3 were stained with monoclonal rat anti-mouse CD68 (1/100 = 1 µg/mL) to detect macrophages. TRITC-conjugated goat anti-rat immunoglobulins (Ig) were used as secondary antibodies (1/200).
(TIF)

Materials and Methods S1 (DOC)
