# Supplementary figures and images for: Coagulation Factor X Interaction with Macrophages through Its N-Glycans Protects It from a Rapid Clearance
Source: PLoS One. 2012 Sep 25;7(9):e45111. doi: 10.1371/journal.pone.0045111 (PMC3458019; doi:10.1371/journal.pone.0045111)

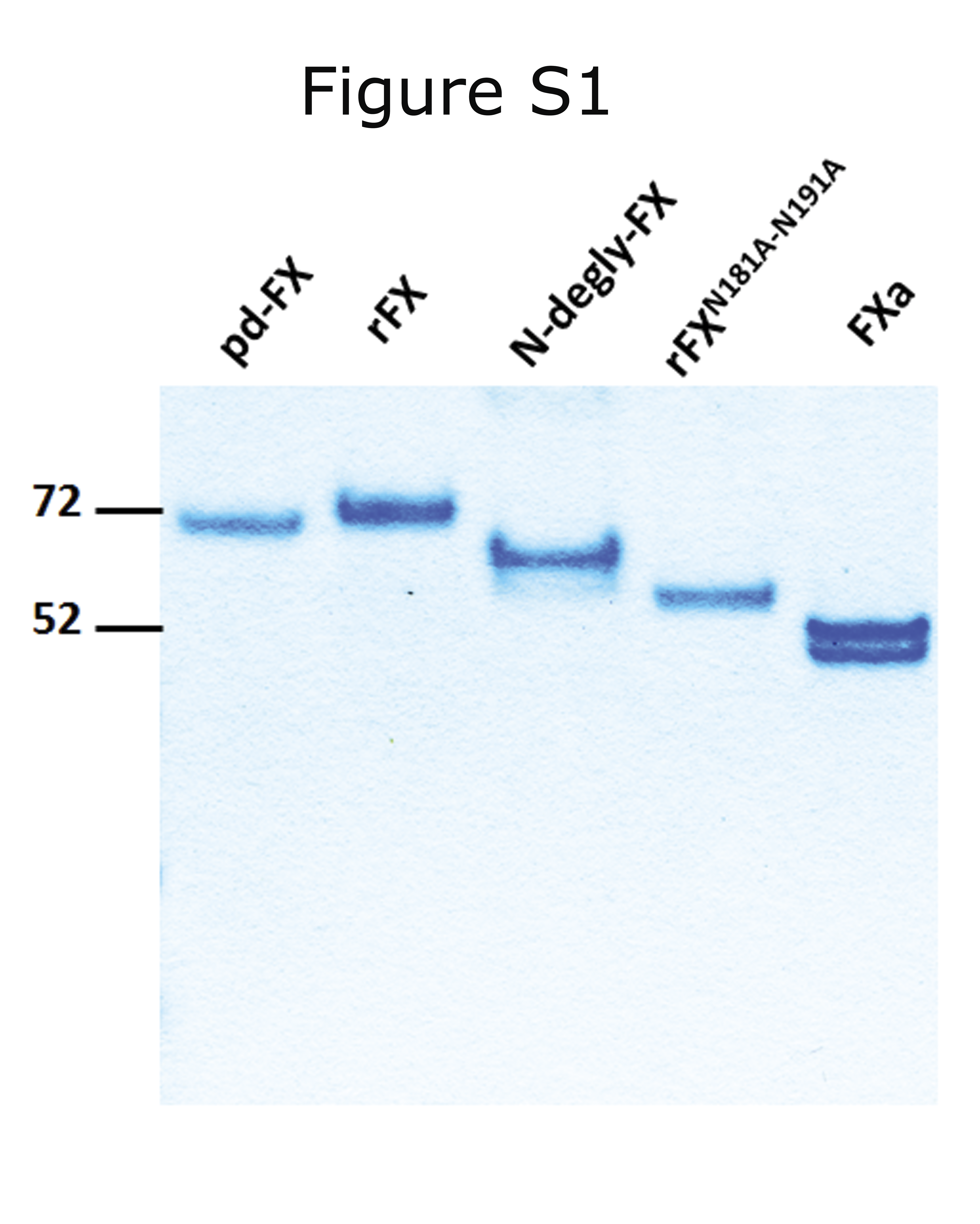

Supplement: Figure S1 — SDS PAGE Analysis of pd-FX, FXa, and N-degly-FX. Purified proteins (2 µg/lane) were subjected to 15% SDS-polyacrylamide gel electrophoresis analysis under non-reducing conditions followed by staining with Coomassie Brilliant Blue R-250. Standards with apparent molecular weights are indicated on the left of the gel; Lane 1, pd-FX; lane 2, rFX; lane 3, N-degly-FX; and lane 4, rFXN181A–N191A, and lane 5 FXa. N-degly-FX was obtained after digestion of pd-FX by PNGase F. rFX and rFXN181A–N191A contain at their carboxy-terminal end a supplementary sequence (EQDDPRLIDGK) recognized by monoclonal antibody HPC4 as previously reported [13], [16]. rFXN181A–N191A corresponds to the FX/AP176–194-N181A–N191A and thus lacks the C-terminus of the activation domain [13]. (TIF) [file pone.0045111.s001.tif]

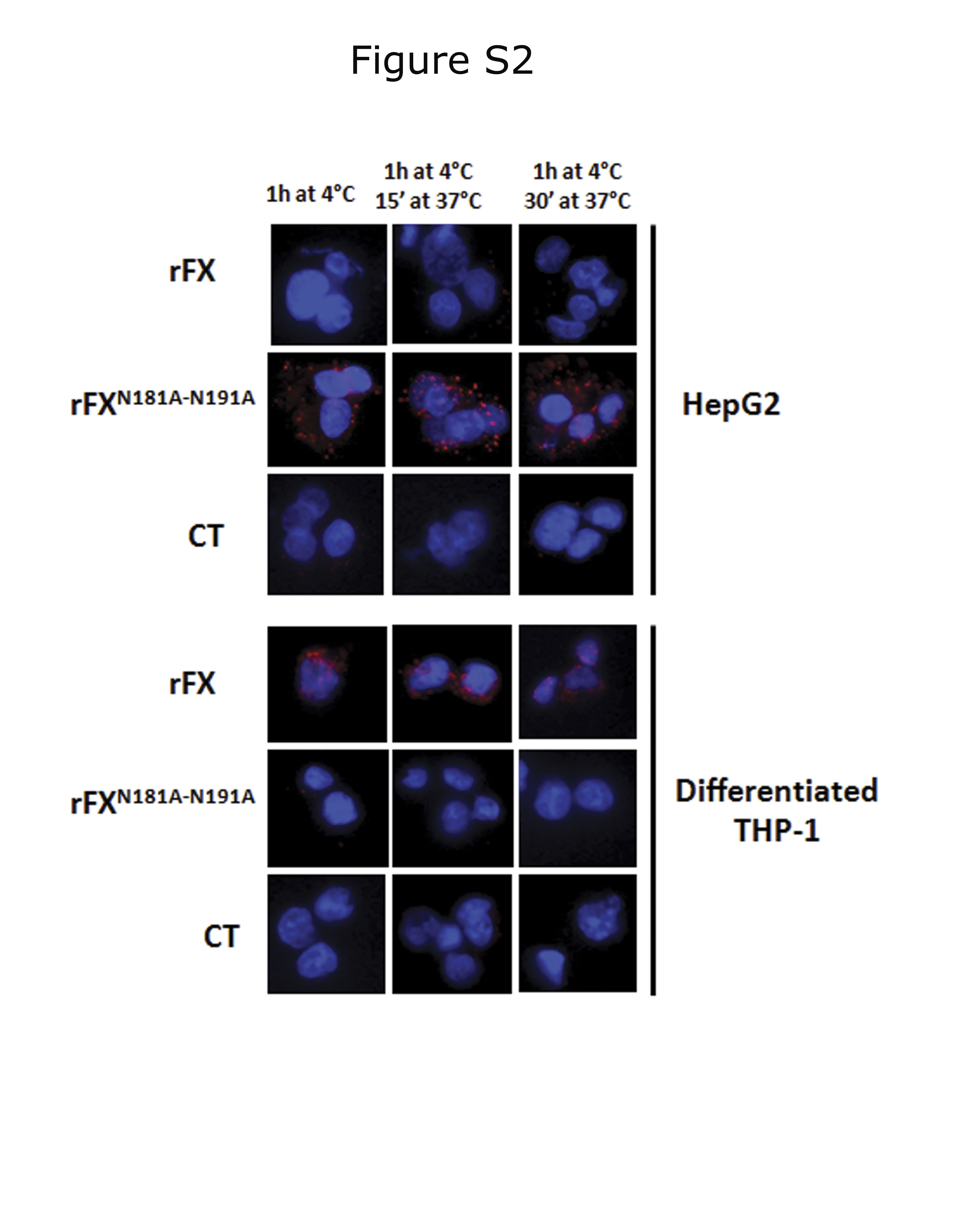

Supplement: Figure S2 — Binding and internalization of rFXN181A–N191A by HepG2 cells and rFX by differentiated THP-1. HepG2 cells and THP-1 cells differentiated to macrophages by PMA (see Materials and Methods) were incubated with 10 µg/mL of rFX, rFXN181A–N191A or with PBS as control (CT) for 1 h at 4°C. Then, after washing cells were incubated at 37°C for 15 and 30 min. Nucleus/DNA was stained with DAPI (blue) and FX variants were visualized by red fluorescence using mouse monoclonal and rabbit polyclonal antibodies both anti-human FX in a proximity ligation assay (PLA)–based method. (TIF) [file pone.0045111.s002.tif]

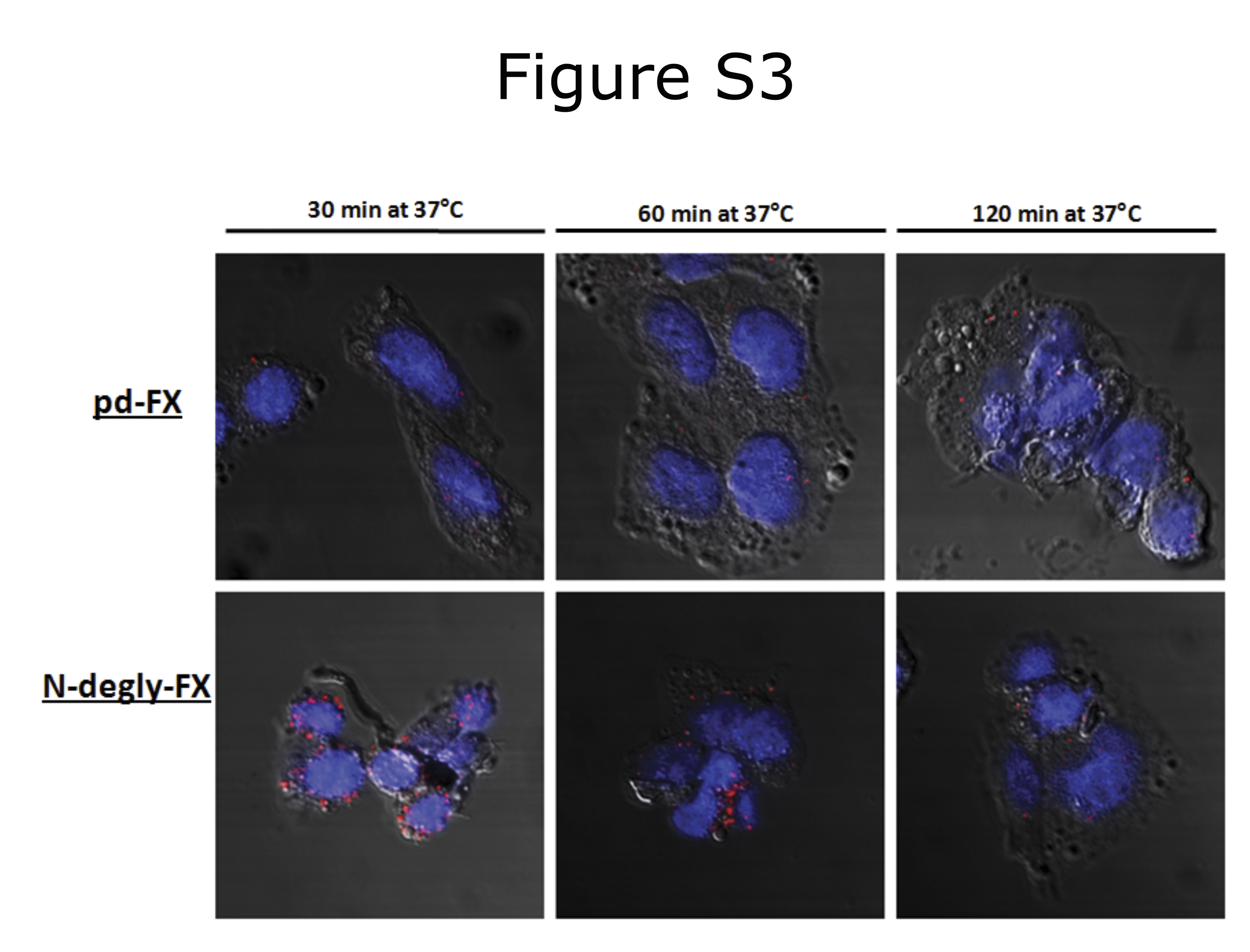

Supplement: Figure S3 — Investigation of the co-localization of pd-FX and N-degly-FX with early endosomes in HepG2 cells busing high-resolution confocal images. HepG2 cells were incubated with 10 µg/mL of pd-FX, N-degly-FX or with PBS as control (CT) for 1 h at 4°C. Then, after washing cells were incubated at 37°C for 30, 60 and 120 min at 37°C. Nucleus/DNA was stained with DAPI (blue) and FX variants were co-localized by red fluorescence using goat anti-human FX with anti-early endosome-antigen 1 in a proximity ligation assay (PLA)–based method. (TIF) [file pone.0045111.s003.tif]

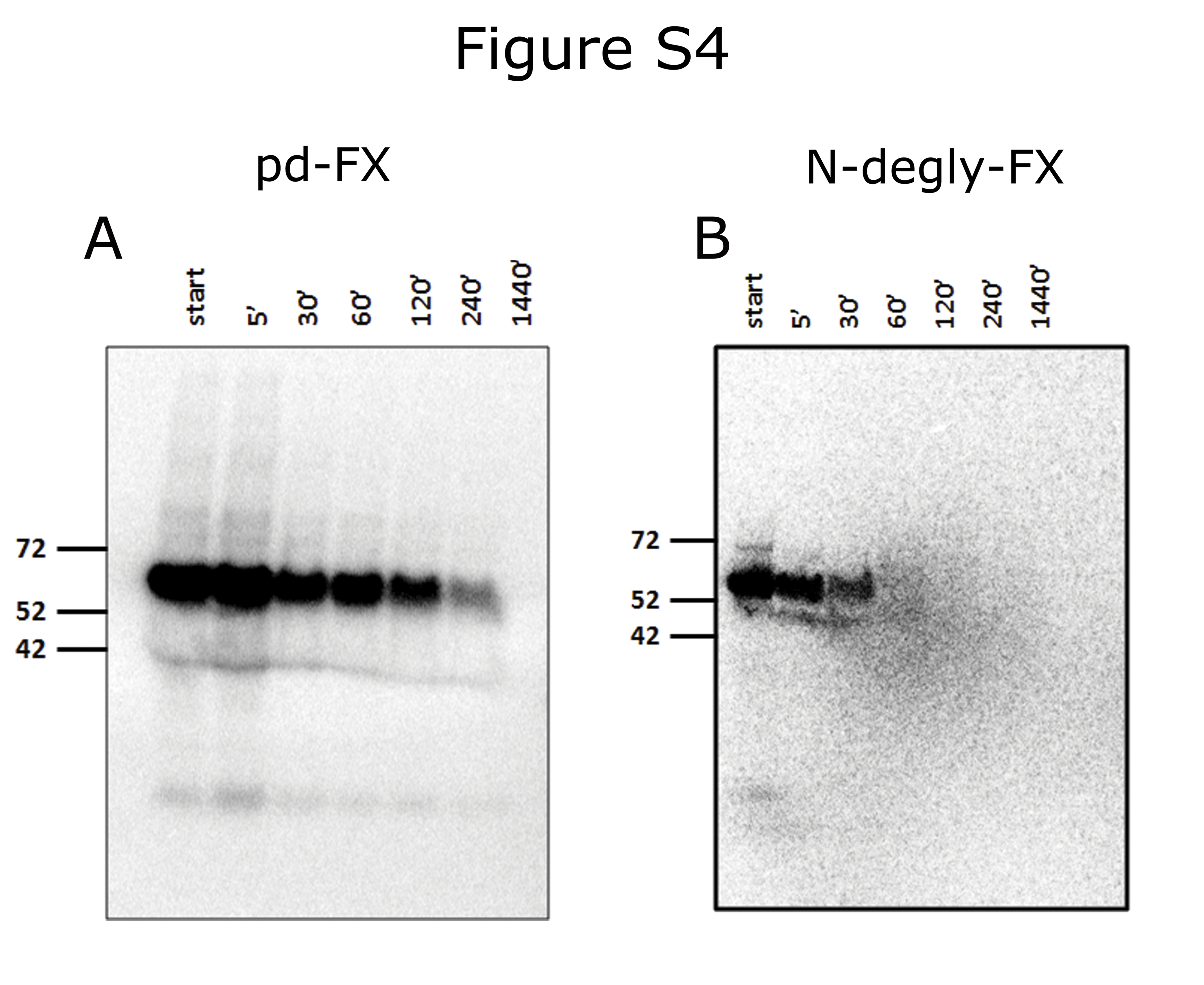

Supplement: Figure S4 — Investigation of radiolabeled FX variants degradation in mouse plasma. Mice were injected with either (A) 125I-pd-FX or (B) 125I-N-degly-FX (10 µg/mouse) and at different time points (5, 30, 60, 120, 240, and 1440 minutes) blood samples were taken. Plasma samples were migrated on 15% SDS-polyacrylamide gel electrophoresis analysis under non-reducing conditions. Results were visualized by autoradiography using PharosFX™ Plus Molecular Imager (BioRad, Hercules, CA, USA). (TIF) [file pone.0045111.s004.tif]

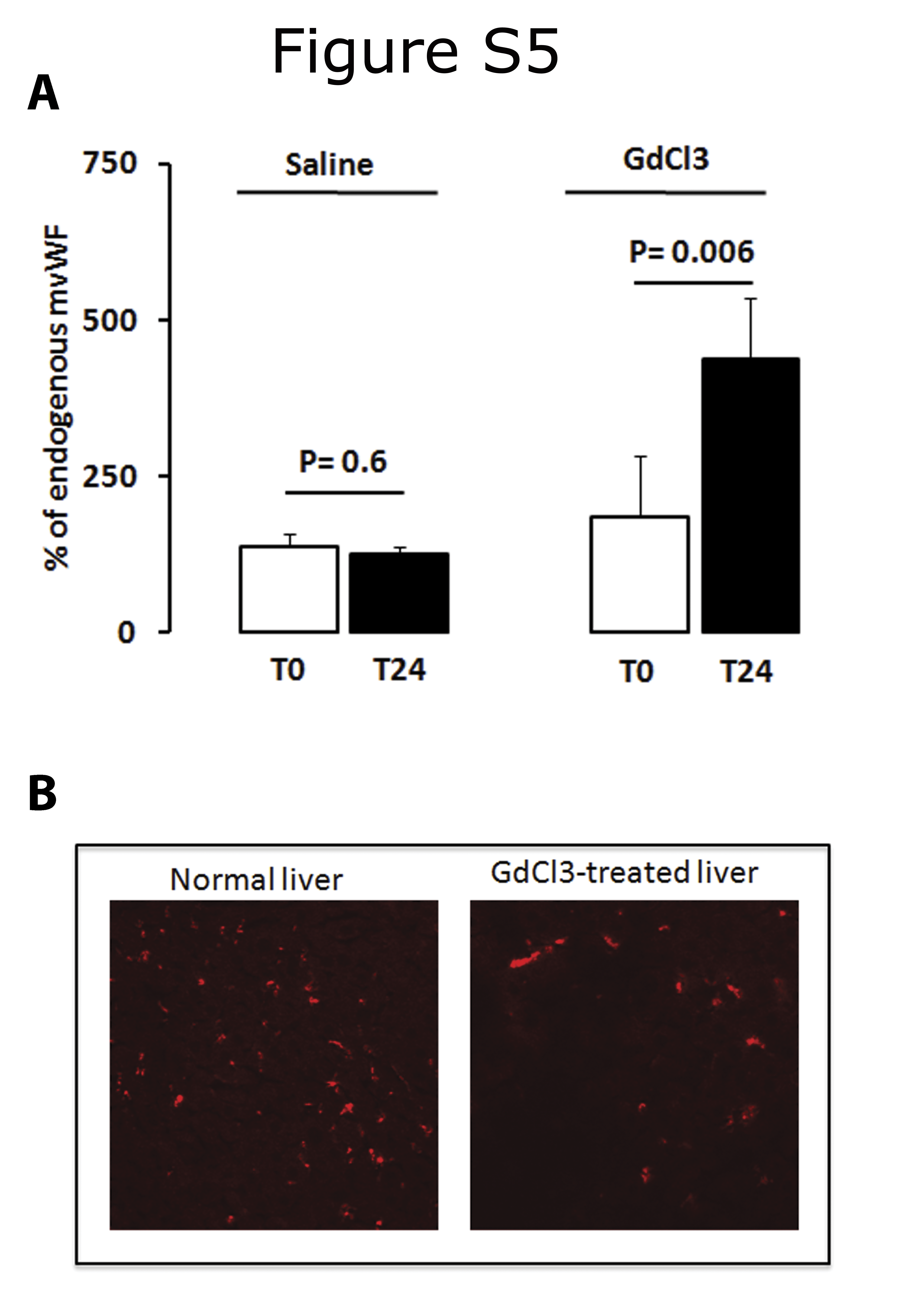

Supplement: Figure S5 — Increased of endogenous VWF levels upon gadolinium chloride treatment. (A) Mice were treated with saline or GdCl3, and 24 hours after treatment (T24) endogenous VWF (mvWF) was measured by ELISA and compared to mvWF levels before (T0) saline or GdCl3 treatment. Data represent mean ± S.D. of 3 experiments. (B) Liver sections of wild-type mice treated with saline (normal liver) or GdCl3 were stained with monoclonal rat anti-mouse CD68 (1/100 = 1 µg/mL) to detect macrophages. TRITC-conjugated goat anti-rat immunoglobulins (Ig) were used as secondary antibodies (1/200). (TIF) [file pone.0045111.s005.tif]
