# Supplementary material for: Coagulation Factor X Interaction with Macrophages through Its N-Glycans Protects It from a Rapid Clearance
Source: PLoS One. 2012 Sep 25;7(9):e45111. doi: 10.1371/journal.pone.0045111 (PMC3458019; doi:10.1371/journal.pone.0045111)
Supplement: Materials and Methods S1 — (DOC) [file pone.0045111.s006.doc]

**Supporting Information**

***Coagulation factor X interaction with macrophages through its N-glycans protects it from a rapid clearance***

***Kurdi et al.***

*Supporting Information Materials & Methods*

### Reagents

Russell’s viper venom X (RVV-X) and chromogenic substrate S-2765 were purchased from Chromogenix (Mölndal, Sweden). Rabbit anti-human FX polyclonal antibodies, conjugated or not with horseradish peroxidase were purchased from Eurogentec (Angers, France). Labeled streptavidin-biotin system-HRP kit was purchased from Dako (Trappes, France). Red blood cell lysis solution (10X), CD11b-MicroBeads, and autoMACS running buffer-MACS separation buffer were provided from Miltenyi Biotec (Bergisch Gladbach, Germany). Collagenase D and DNase I was purchased from Sigma Aldrich (Saint-Quentin-en-Fallavier, France). HiTrap affinity columns were from GE Healthcare (Orsay, France). SignalStain® Boost IHC Detection Reagent (HRP, Rabbit) was purchased from Cell Signaling Technology (Boston, MA, USA).

### Preparation of factor X without N-glycans

Full-length Glycosidase digestion experiments under mild conditions on N-linked glycans of pd-FX were carried out using PNGase F from Chryseobacterium as previously described.[1] Briefly, 100 µg of pd-FX was diluted with PNGase F (25.000 U/mL) in 0.05 M sodium phosphate, pH 7.5, 1% NP-40 and incubated at 37 °C for 2 h. Then, the treated protein was purified from PNGase F by ion exchange chromatography on *GE Healthcare* Mono Q *HR 5/5 column and dialyzed against PBS* overnight at 4 °C. Protein purity (>95%) and full removal of N-glycans was assessed using 15% SDS-polyacrylamide gel electrophoresis analysis under non-reducing conditions followed by staining with Coomassie Brilliant Blue R-250 (see Supporting Information: figure S1).

*Production of recombinant human FX and mutated at N-glycan sites*

The two constructs, recombinant wild-type FX (rFX) and recombinant FX mutated at both N-glycosylation sites (rFX^N181A-N191A^), previously described[2] were produced in BHK-21 cells and purified as reported.[3] Prior to use, a final pass over a benzamidine Sepharose column was used to eliminate any trace of FXa potentially generated during production or purification. Protein purity (>95%) was assessed using 15% SDS-polyacrylamide gel electrophoresis analysis of the FX derivatives under non-reducing conditions followed by staining with Coomassie Brilliant Blue R-250 (see Supporting Information: figure S1).

Quantification and characterization of plasma derived and recombinant FX preparations

ELISA using rabbit polyclonal antibodies against human FX conjugated or not with horseradish peroxidase assayed all FX preparations (FX:Ag). Similar affinities were found between pd-FX, N-degly-FX, rFX, and rFX^N181A-N191A^ for rabbit anti-human FX polyclonal antibodies. Furthermore, the activity of the FX preparations (FX:RVV-X activity) was determined by an amidolytic assay using RVV-X (Hyphen Biomed, Neuville-sur-Oise, France). In these two assays, a commercial pool of normal human plasma was used as standard. This pool was estimated to contain 10 µg/ml of human FX. Moreover, a purified batch of pd-FX from Haematologic Technologies Inc of known concentration was used as internal control. For all the FX preparations used in this study, the two assays gave similar values (ratio FX:Ag/FX:RVV-X activity = 1.0 ± 0.2).

*Tissue collection*

Mice were injected intravenously in the tail-vein with pd-FX, N-degly-FX, rFX or rFX^N181A-N191A^ diluted at 10 µg/mL in PBS. Mice were bled, anesthetized and killed. Then, tissue was collected and perfused with PBS. For paraffin sections, individual lobes of mouse livers were embedded in paraffin wax blocks. Paraffin sections were cut and mounted on Superfrost plus glass slides before dewaxing in xylene and rehydration in a serial ethanol gradient. For cryostat sections, mouse livers were perfused with PBS, embedded in Tissue-Tek OCT-compound (miles laboratories, Elkhart, IN), and immediately frozen in liquid nitrogen and maintained at -20°C. Tissue was cut into sections on a freezing microtome (Leica CM3050S, Leica MicroSystems SAS, Nanterre, France) and mounted on slides. Sections were washed in PBS and permeabilized with triton 0.5% in PBS. Then, for paraffin sections, endogenous peroxidase was quenched by incubating the sections for 10 min in 3% H_2_O_2_ at RT. Then, cryostat and paraffin sections were incubated with bovine serum albumin 3% (BSA) in PBS containing murine IgG (1/500 = 2.5 µg/mL) to saturate non-specific section surfaces and Fc receptors.

### Histological analysis of mice livers

Using paraffin sections, antigens were localized by incubation for one hour at room temperature with rabbit anti-human FX (1:1000 dilution = 3.37 µg/mL) primary antibody. Sections were washed three times in PBS, incubated with SignalStain® Boost IHC Detection Reagent (HRP, Rabbit) for 30 min at room temperature. After washing in PBS, diaminobenzamidine (DAB) (Dako LSAB 5001) was added to allow visualization of the bound antibody. The tissue sections were washed in PBS, and slightly counterstained with diluted hematoxylin in order to allow a better localization of the labeled signal.

Cryostat sections were immunostained using monoclonal rat anti-mouse CD68 (1/100 = 1 µg/mL) to detect macrophages. TRITC-conjugated goat anti-rat immunoglobulins (Ig) were used as secondary antibodies (1/200). FX was detected by using polyclonal rabbit anti-human FX (Eurogentec 1/1000 = 3.37 µg/mL) and antibodies were revealed by AF488-conjugated goat anti-rabbit Ig as secondary antibodies (1/1000).

### Isolation and purification of mice liver cells

**-** Hepatocytes were isolated by two-steps perfusion technique as previously described[4] derived from the Seglen technique.[5] Briefly, mice were anesthetized by a mix Ketamine/Xylasine. Mouse liver was first perfused at 4 mL/min with pre-heated HEPES buffer at 42°C (2-[4-(2-hydroxyethyl)-1-piperazinyl]) and then with HEPES buffer containing collagenase D (4 mg/mL) and CaCl_2_ (5 mM). After perfusion, homogenous liver cell suspension that contained hepatocytes was obtained by gentle mechanical dispersion in DMEM/F12 medium containing 10% FBS, 1% glutamate, 1% BSA and 1% penicillin/streptomycin. Cells were washed and centrifuged at 800 g for 4 times with the same medium. Finally, cells were maintained in collagen pre-coated Petri-dishes for 4 h within an incubator at 37°C with 5% CO_2_. Hepatocytes viability was estimated by Trypan Blue incorporation. Cells were used only when the viability exceed 90% and within 6 hours after their isolation from healthy animals without hepatitis or viruses which are crucial parameters to preserve the viability and functionality of hepatocytes.[6-8]

**-** For Kupffer cells isolation, mouse liver was dissected and non-parenchymal mouse liver cell suspension was prepared using the gentle MACS dissociator as described by the provider (Miltenyi Biotech). Briefly, the liver was transferred into a GentleMACS tube with a pre-warmed solution at 37°C of KRB (Krebs Ringer Buffer) containing CaCl_2_ (20 mM), MgCl_2_ (20 mM), collagenase D (final concentration 500 CDU/mL), and DNase I (final concentration 150 U/mL). The product was incubated for 30 min at 37°C and the dissociation step repeated. Then, the liver solution was applied onto cell strainer (70 µm mesh size) and suspended with PEB buffer (PBS, 0.5% BSA, and 2 mM EDTA). Cell suspension was centrifuged at 21 g for 4 min at 4°C and supernatant was collected and resuspended in PEB buffer and centrifuged at 300 g for 10 min at 4°C. Cell pellet was resuspended in PEB buffer, incubated for 5 min at RT in the red blood cell lysis solution (Miltenyi Biotech), and then rewashed by PEB buffer.

For purification of Kupffer cells, cell pellet was resuspended in PEB buffer and was applied onto cell strainer (30 µm mesh size), centrifuged at 300 g for 10 min at 4°C. Cell pellet was resuspended in 80 µL of PEB buffer and incubated for 15 min at 4-8°C in the presence of 20 µL of CD11b-MicroBeads (Miltenyi Biotech) per 10^7^ cells. The selection of CD11b positive cells to isolate murine Kupffer cells is also a way to deplete them from sinusoidal endothelial cells as previously described by others using the same approach.[9,10] Cells were washed by adding PEB buffer and centrifuged at 300 g for 10 min at 4°C. Cell pellet was resuspended in 500 µL PEB buffer. CD11b positive cells were purified by magnetic separation with MS columns (Miltenyi Biotech) and characterized by monoclonal rat anti-mouse CD68 (1/100 = 1 µg/mL). TRITC-conjugated goat anti-rat immunoglobulins (Ig) were used as secondary antibodies (1/200). Purified cells were maintained in serum free RPMI medium with GlutaMax^TM^ and incubated in 24-well tissue culture plates for 30 min within a CO_2_-incubator at 37°C with 5% CO_2_. Then, supernatant was discarded and RPMI medium with GlutMax^TM^ containing 10% FBS and 1% penicillin/streptomycin was added and cells were incubated overnight within a CO_2_-incubator at 37°C with 5% CO_2_.

*Gadolinium chloride treatment of mice*

The experiment was done as previously described.[11] Briefly, Gadolinium chloride (GdCl_3_, 50 mg/kg of body weight) diluted in 200 µl physiological serum (or 200 µl physiological serum as control) was given to mice via intravenous tail injection 24 hours prior to the administration of 10 µg of purified ^125^I-pd-FX or ^125^I-N-degly-FX diluted in PBS. Depletion of macrophages in the liver due to GdCl_3_ treatment was assessed as previously described[11] and by the observation of a markedly increased of endogenous von Willebrand factor antigen level upon treatment. At 15 min after injection, mice were anesthetized with tribromoethanol (0.15 mL/g body weight), blood was collected by retro-orbital venous sampling on citrate, and the liver was collected. Radioactivity in plasma and liver was measured in a gamma counter, and the percentage of radioactivity present in each tissue was calculated in relation to the total amount of injected radioactivity.

*Microscopic imaging*

Images were visualized using an AxioImager A1 microscope (Carl Zeiss, Göttingen, Germany) using a Plan-Apochromat 63x-NA 1.4 objective or an EC Plan-Neofluor 40x/0.75 air objective (Carl Zeiss). For quantification, 5 to 35 images of cells per condition were randomly collected and analyzed employing depending of the technique used for visualization either ImageJ-1.44 software (htpp://rsbweb.nih.gov/ij/index.html) or BlobFinder v3.2. software package (Olink Bioscience, Uppsala, Sweden). Confocal images were obtained using an Axiovert 200M microscope using a Plan-Apochromat 63x-NA 1.4-oil immersion objective and a Zeiss LSM510-meta confocal system (Carl Zeiss). The acquisition software was AxioVision LE 7.7.1 (Carl Zeiss). Scanning of the microscope slides was done using Scanscope CS apparatus (TRIBVN licensing APERIO, Chatillon, France).

### Statistical analysis

Data are expressed as mean values plus or minus SD, unless indicated otherwise. Statistical analyses of continuous parameters were performed using the Student unpaired *t* test. *P*-values less than 0.05 were considered statistically significant.

*References of the Supporting Information*

1. Liu-Chen LY, Chen C, Phillips CA (1993) Beta-[3H]funaltrexamine-labeled mu-opioid receptors: species variations in molecular mass and glycosylation by complex-type, N-linked oligosaccharides. Mol Pharmacol 44: 749-756.

2. Gueguen P, Cherel G, Badirou I, Denis CV, Christophe OD (2010) Two residues in the activation peptide domain contribute to the half-life of factor X in vivo. J Thromb Haemost 8: 1651-1653.

3. Levigne S, Thiec F, Cherel G, Irving JA, Fribourg C, et al. (2007) Role of the alpha-helix 163-170 in factor Xa catalytic activity. J Biol Chem 282: 31569-31579.

4. Overturf K, Al-Dhalimy M, Tanguay R, Brantly M, Ou CN, et al. (1996) Hepatocytes corrected by gene therapy are selected in vivo in a murine model of hereditary tyrosinaemia type I. Nat Genet 12: 266-273.

5. Seglen PO (1976) Preparation of isolated rat liver cells. Methods Cell Biol 13: 29-83.

6. Nishitai R, Koch CA, Ogata K, Knudsen BE, Plummer TB, et al. (2005) Toward the survival and function of xenogeneic hepatocyte grafts. Liver Transpl 11: 39-50.

7. Hamel F, Grondin M, Denizeau F, Averill-Bates DA, Sarhan F (2006) Wheat extracts as an efficient cryoprotective agent for primary cultures of rat hepatocytes. Biotechnol Bioeng 95: 661-670.

8. Weber A, Groyer-Picard MT, Franco D, Dagher I (2009) Hepatocyte transplantation in animal models. Liver Transpl 15: 7-14.

9. Do H, Healey JF, Waller EK, Lollar P (1999) Expression of factor VIII by murine liver sinusoidal endothelial cells. J Biol Chem 274: 19587-19592.

10. Kumar V, Ali SR, Konrad S, Zwirner J, Verbeek JS, et al. (2006) Cell-derived anaphylatoxins as key mediators of antibody-dependent type II autoimmunity in mice. J Clin Invest 116: 512-520.

11. van Schooten CJ, Shahbazi S, Groot E, Oortwijn BD, van den Berg HM, et al. (2008) Macrophages contribute to the cellular uptake of von Willebrand factor and factor VIII in vivo. Blood 112: 1704-1712.
